# Chemical and Ultrastructural Changes in the Cuticle Observed in *RabA2b* Overexpressing Plants

**DOI:** 10.3390/plants15030408

**Published:** 2026-01-29

**Authors:** Opal Bechar, Sanaa Musa, Boris Fichtman, Ifat Matityahu, Yehoram Leshem

**Affiliations:** 1 MIGAL—Galilee Research Institute, Kiryat Shmona 1101602, Israelsanaa@migal.org.il (S.M.);; 2 Tel-Hai University of Kiryat Shmona and the Galilee, Kiryat Shmona 1101602, Israel; 3 Azrieli Faculty of Medicine, Bar-Ilan University, Safed 1311502, Israel; bfichtman@gmail.com

**Keywords:** cuticle, plasma membrane trafficking, RabA small GTPases, drought stress

## Abstract

The plant cuticle is a hydrophobic layer covering the cell wall that protects cells from pathogen invasion and water loss. In this study, we analyzed the cuticles of transgenic *Arabidopsis thaliana* plants overexpressing the vesicular trafficking small GTPase *RabA2b*. The *RabA2b*-overexpressing plants exhibited distinctive structural and chemical modifications in their cuticles, including enhanced hair-like wax crystals and increased accumulation of phenolic compounds such as ferulic acid and coumaric acid, which contribute to cutin cross-linking and reinforcement of the cuticle matrix. These chemical and structural changes were associated with improved barrier function and increased drought resistance. Our findings suggest the involvement of *RabA2b* in affecting the plant cell’s exterior by altering the cuticle composition and architecture, thereby improving plant tolerance to water deficit.

## 1. Introduction

Terrestrial plants are constantly losing water to the environment. During their evolution on land, plants developed various epidermal characteristics, which assist in retaining their endogenous water. These characteristics include tight regulation of transpiration through the stomatal pore that closes upon water limitation conditions [1,2]. In addition, an extracellular hydrophobic layer, named the cuticle, was developed, and is formed beyond the cell wall, providing further protection against non-stomatal water loss [3]. The cuticle can also play a role in other biological functions, such as protection against radiation and various bacteria, fungi, and insects [4].

The plant cuticle is composed mainly of cutin and wax. Cutin is a lipid polyester built predominantly from oxygenated C16 and C18 fatty acids and glycerol, but it can also incorporate phenolic derivatives originating from the phenylpropanoid pathway, such as ferulic and *p*-coumaric acids. These phenolic compounds can be esterified into cutin monomers and contribute to the architecture of the cutin matrix [5]. Crosslinking of ferulate and coumarate strengthens the cutin polymer network, producing a denser and more rigid barrier that reduces cuticular water loss and enhances drought tolerance [6,7,8]. Although ferulic acid is a major monomer of suberin in *Arabidopsis thaliana*, several studies indicate that it may also be incorporated into cutin in different plant species. In contrast to cutin, the cuticular wax consists mainly of very-long-chain fatty acids (typically C20–C34) and their derivatives, alkanes, aldehydes, alcohols, ketones, and esters, which form the hydrophobic outer barrier that also contributes to limiting non-stomatal water loss [9]. The wax is both dispersed within the cutin (intra-cuticle) and deposited on top of the cutin in a distinct layer (epi-cuticle) [10]. In many cases, the epi-cuticular wax layer is crystalline. The morphology of epi-cuticular wax crystals, whether plate-like, rod-shaped, or tubular, is largely determined by wax chemical composition (chain-length distribution and functional groups), the relative abundance of specific wax classes, and environmental or genetic factors that influence their self-assembly on the cuticle surface [11]. Recent studies showed that in spite of its hydrophobic nature, significant amounts of water may be lost from the plant through its cuticle [12,13].

Since most cuticle precursors are synthesized in the endoplasmic reticulum (with the exception of phenylpropanoid-derived precursors produced in the cytosol), cuticle biosynthesis depends on trafficking, transport, and secretion mechanisms that deliver these compounds to the cell surface [14,15,16]. Plant cell secretion is known to be mediated by vesicle trafficking. Amongst the proteins known to play a key role in vesicle trafficking regulation are Rab small GTPases [17]. The Rab superfamily in *Arabidopsis thaliana* consists of 57 members that are divided into 8 subfamilies (A–H). The different Rabs are localized to distinct intracellular membranes and mediate the multiple steps along the exocytic and endocytic pathways. The RabA subfamily has been shown to be involved in plasma membrane (PM) trafficking and cell wall (CW) metabolism [18,19].

Recently, we reported that overexpressing RabA2b in *Arabidopsis thaliana* significantly improves plant drought tolerance [20]. We also demonstrated by the Toluidine blue assay that the leaves of the RabA2b overexpressing plants were less permeable to water as compared to wild-type (WT) leaves. In addition, we found through a proteomic study that the plasma membrane of the transgenic plants was enriched with several cuticle metabolism-related proteins (such as GDSL, LTP5, and SQE3). Taken together, these findings suggested that the cuticle of the transgenic plants was altered. In the current study, we investigated the impact of RabA2b overexpression on the cuticle chemical composition, including phenolic acid content, and its structural features.

## 2. Results

### 2.1. Chemical Changes in the Cuticle of RabA2b Over-Expressing Plants

**Figure 1:**
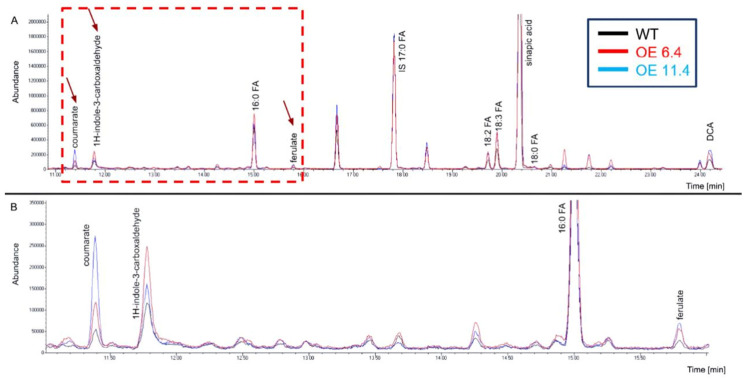
Identification and quantification of Cutin polyester monomers in *Arabidopsis* leaves. (**A**) Representative GC-MS chromatogram of Cutin polyester monomers identified in leaf extractions of WT and two transgenic lines overexpressing *RabA2b* (6.4 and 11.4). (**B**) Enlargement of the red area boxed in A. Red arrows in A mark the peaks corresponding to coumarate, 1H-Indole-3-carboxaldehyde and ferulate, which are enlarged at B. The polyester monomers were extracted as described earlier by Jenkin and Molina (2015) [9].

To examine and compare the chemical composition of the cuticle in WT and transgenic plants overexpressing (OE) *RabA2b* (lines OE 6.4 and OE 11.4), the tested genotypes were grown in standard conditions. Their rosette leaves were collected, and the cutin polymers were isolated and depolymerized to monomers as previously described [9]. The samples were then analyzed by GC-MS, which identified and quantified eight major compounds typically associated with the cuticular polyester matrix, based on their retention times (RT) (Figure 1 and Table 1). No significant differences were detected in the total levels of the major fatty acid monomers among the tested genotypes. However, clear quantitative differences were observed in several metabolites.

The phenolic compounds ferulate (ferulic acid), coumarate (coumaric acid), and 1H-indole-3-carboxaldehyde (I3CA) were significantly elevated in both OE lines compared with WT. Ferulate (RT = 15.80 min) showed the most pronounced increase, rising approximately 1.55-fold in both OE 6.4 and OE 11.4 lines (*p* ≤ 0.0001 and *p* ≤ 0.001, respectively). Coumarate (RT = 11.39 min) levels were 1.36-fold higher in OE 6.4 and 1.59-fold higher in OE 11.4 (*p* ≤ 0.05). Similarly, I3CA (RT = 11.78 min) also accumulated to a greater extent, increasing 1.27-fold and 1.28-fold in OE 6.4 and OE 11.4, respectively (*p* ≤ 0.01 and *p* ≤ 0.05). In contrast, the levels of the major fatty acid monomers, palmitic acid (16:0 FA), linoleic acid (18:2 FA), linolenic acid (18:3 FA), and stearic acid (18:0 FA), remained largely unchanged (fold change ≈ 1.0; *p* > 0.05). Sinapic acid (RT = 20.35 min) also showed no significant variation among genotypes (Table 1).

### 2.2. Ultrastructural Changes in the Cuticle of RabA2b Over-Expressing Plants

Arabidopsis inflorescence stems produce 10- to 20-fold more total wax per area than leaves [10,21]. The stem’s epicuticle surface is highly complex and consists of a variety of wax crystal structures of different sizes and shapes, which are undetected on the leaf surface [21]. Therefore, the stem is commonly used as a model tissue for epicuticle wax studies [14,22].

To analyze the morphology and topography of the epicuticular surface, we used Scanning Electron Microscopy (SEM) to examine the stems of WT and *RabA2b* OE plants. Inflorescence stem samples were vapor-fixed in the presence of aqueous solutions of paraformaldehyde (16%), glutaraldehyde (8%), and osmium tetroxide (4%), air-dried, coated with ~5 nm iridium, and visualized by SEM as described elsewhere [23].

**Figure 2:**
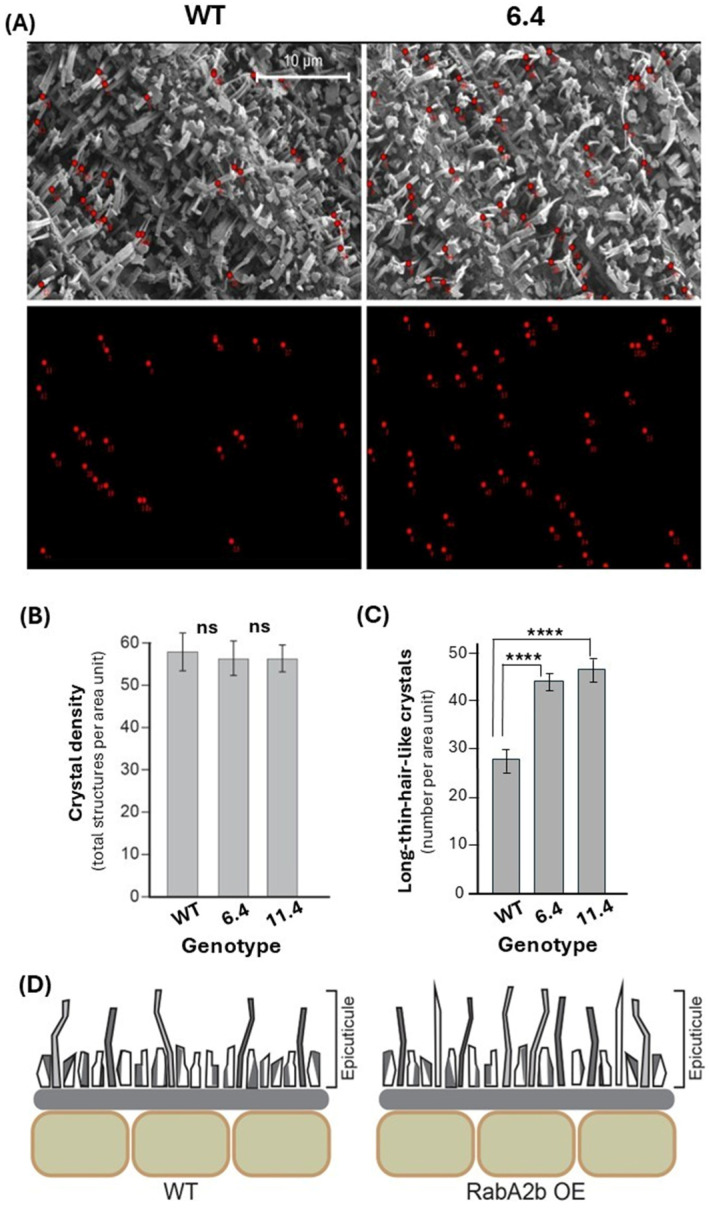
SEM analysis of the epicuticular topography in Arabidopsis stems. (**A**) Representative SEM micrographs of epicuticular wax crystals of WT and transgenic lines overexpressing *RabA2b* (6.4 and 11.4). Red dots in the upper and lower panels indicate the long-thin hair-like epicuticular crystal waxes. (**B**) Quantification of the total number of epicuticular structures (number per area). (**C**) Quantification of the long-thin hair-like epicuticular crystal waxes presented in A. (**D**) Graphic representation of the epicuticular topography phenotype in WT and *RabA2b* overexpressing plants. In B and C, three SEM micrographs were captured per genotype and divided into four fields of 934 μm^2^ each, and the red dots presented in A were counted manually. Asterisks (****) represent significant *p*-values at *p* ≤ 0.0001 (Student’s *t*-test). Error bars are standard deviations, ns—not significant.

High-resolution SEM images of the stem epicuticular surface revealed a complex morphology, characterized by wax crystals of various sizes and shapes, including hair-like, columnar-shaped rods, vertical plates, dendritic, and umbrella-like structures (Figure 2A and Figure S1). This complex surface morphology was observed in all of the tested genotypes. The total density of these structures (per unit area) was similar across the tested genotypes (Figure 2B). However, the abundance of the long, thin hair-like crystals was significantly higher in the *RabA2b* OE plants. Quantification of these structures per unit area indicated a 70% increase in their abundance in the transgenic lines compared with the WT (Figure 2C,D).

## 3. Discussion

**Figure 3:**
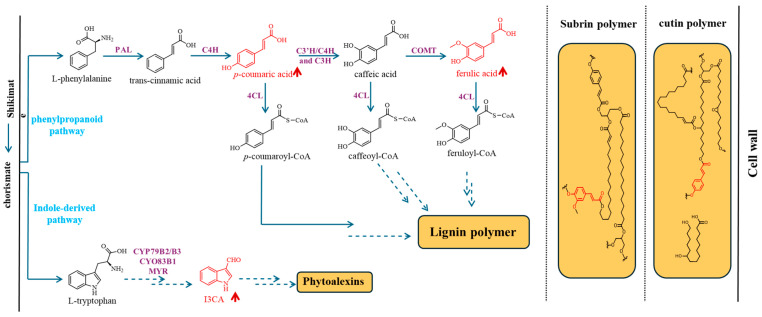
Schematic representation of two metabolic pathways: the phenylpropanoid and indole-derived pathways. Dashed arrows indicate multiple intermediate steps. I3CA: indole-3-carboxaldehyde. Key enzymes shown include PAL (phenylalanine ammonia-lyase), 4CL (4-coumarate: CoA ligase), C3′H/C4H/C3H (p-coumarate 3-hydroxylase/cinnamate 4-hydroxylase/coumarate 3-hydroxylase), COMT (caffeic acid O-methyltransferase), and MYR (myrosinase). Metabolites shown in red are upregulated in *RabA2b* OE plants. On the right side, highlighted in red are the positions where phenolic compounds integrate into the Cutin and Suberin backbones.

The accumulation of ferulate, coumarate, and indole-3-carboxaldehyde (I3CA) in the cuticle of *RabA2b* OE plants suggests that RabA2b-mediated trafficking impacts multiple secondary metabolic pathways, which have been documented in previous studies [15,24,25,26]. The major pathways associated with the upregulated metabolites are illustrated in Figure 3.

One major pathway affected is the phenylpropanoid pathway, which originates from phenylalanine. Phenylalanine is deaminated by phenylalanine ammonia lyase (PAL) to yield cinnamate, which is subsequently hydroxylated by cinnamate 4-hydroxylase (C4H) to form *p*-coumarate. Through further hydroxylation, methylation, and oxidation steps, *p*-coumarate is converted to ferulate. Both *p*-coumarate and ferulate can serve as precursors for the biosynthesis of cutin and suberin, where they act as ester-linked phenolic monomers that reinforce the polymeric network, as well as for the lignin biosynthesis pathway. Even in the absence of changes in fatty acid composition, increased incorporation of ferulate and *p*-coumarate into the cuticle can enhance cross-linking, strengthening the physical barrier and improving its structural rigidity and hydrophobicity [5]. These changes reinforce protection against dehydration and pathogen invasion, thereby contributing to enhanced drought tolerance.

Ferulic acid is a well-established monomeric component of the polyesters cutin and suberin [7,9]. Liu et al. demonstrated that a mutation in *Arabidopsis* *ALDH2C4* (which encodes coniferaldehyde dehydrogenase, which oxidizes coniferaldehyde to ferulic acid) resulted in decreased ferulic acid content and increased drought sensitivity [27]. The elevated ferulate content and enhanced drought tolerance observed in *RabA2b* OE plants are thus consistent with this finding, underscoring a potential role for ferulic acid in mediating plant responses to water deficit.

*RabA2b* OE plants also accumulate higher levels of coumarate, which precedes ferulate in the lignin biosynthesis pathway (which initiates from phenylalanine) (Figure 3) [15]. The concurrent increase in both metabolites suggests that *RabA2b* overexpression may enhance the lignin pathway, potentially increasing lignin content, an aspect to be examined in future studies. Notably, no significant changes were detected in the fatty acid components of cutin and wax, indicating that *RabA2b* overexpression may selectively influence the trafficking or incorporation of phenolic compounds into the cuticle rather than bulk lipid synthesis. This aligns with the established role of *RabA2b* in vesicle-mediated transport to the plasma membrane, which could preferentially modulate phenolic deposition within the cutin matrix.

The biosynthesis of monomeric precursors of the lignin pathway occurs within the cytoplasm [16]. Given the known involvement of RabA2b in cell wall metabolism [18,19], increased RabA2b-mediated PM vesicle trafficking in the transgenic plants may enhance export of these precursors to the cell surface. Alternatively, RabA2b-mediated trafficking may directly or indirectly affect the activity of key enzymes in the lignin biosynthesis pathway. Previous plasma membrane proteomics revealed enrichment of lipid transfer protein 5 (LTP5, AT3G51600) in the PM of *RabA2b* OE plants [20]. Although the role of lipid transfer proteins remains incompletely understood, growing evidence suggests their involvement in the transfer and deposition of monomers required for assembling hydrophobic barriers, including cutin, cuticular wax, suberin, and sporopollenin [28]. Additionally, the PM of *RabA2b* OE plants was enriched in a GDSL enzyme (AT5G55050), a member of a group with esterase/acyltransferase/lipase activity. Several GDSLs are essential for cutin deposition in tomato fruit cuticles [29] and barley grains [30], and phylogenetic analysis places AT5G55050 within a subclade of cutin-synthase-related GDSLs [31].

A second pathway influenced by RabA2b is indole-derived metabolism, which branches from tryptophan and produces 1H-indole-3-carboxaldehyde (I3CA), a precursor of defense-related phytoalexins with reported antimicrobial activity [32,33,34]. The elevated I3CA levels observed in *RabA2b* OE plants indicate that *RabA2b* overexpression may enhance the trafficking or deposition of indolic defense metabolites into the cuticle without altering the fatty acid backbone of cutin and wax. This enrichment of indole- and phenylpropanoid-derived compounds suggests that RabA2b-mediated vesicle trafficking operates at the interface between cytoplasmic primary metabolism and surface deposition of protective metabolites. This selective modulation of phenolic deposition, rather than fatty acid synthesis, provides a plausible explanation for the observed improvement in drought tolerance of *RabA2b* OE plants and potentially suggests enhanced pathogen resistance for these plants.

In addition to biochemical changes, structural modifications of the cuticle may also contribute to drought tolerance in *RabA2b* OE plants. Increased epicuticular wax crystals have been associated with improved drought resistance in multiple systems, including a CRISPR/Cas9-generated *OsPYL9* rice mutant that accumulated higher ABA, antioxidants, chlorophyll, and wax crystals [35], supporting the established role of epicuticular waxes in limiting water loss. Consistently, we observed an increased abundance of long, hair-like wax crystals on the cuticle surface of *RabA2b* OE plants. Although the precise mechanism by which these structures reduce water loss is not yet resolved, their morphology may influence the tissue microclimate by stabilizing the boundary layer, a thin zone of humid, slow-moving air that restricts water vapor diffusion [36,37,38]. A higher density of elongated wax crystals could maintain boundary-layer thickness against air disturbances, thereby reducing transpiration and contributing to the drought resistance observed in the OE lines [39]. Supporting this structural phenotype, plasma membrane proteomics of *RabA2b* OE plants revealed enrichment of an ABC transporter (At2g47800) [20], a protein family known to mediate epicuticular wax transport [40]. Whether this transporter contributes to the uneven wax distribution and enhanced wax crystal formation in *RabA2b* OE plants remains to be determined in future studies.

## 4. Materials and Methods

### 4.1. Plant Material and Growth Conditions

*RabA2b* overexpressing (OE) *Arabidopsis thaliana* and wild type (WT) Col-0 plants were used in this study. The transgenic plants’ details were described previously ref. [19]. The plants were grown for six weeks under white light (120–130 µmol/m^2^ s) in a growth room with day/night cycles of 16 h at 24 °C and 8 h at 18 °C, with humidity of 40–60%.

### 4.2. Arabidopsis Lipid Polyesters Chemical Analysis

Following a published protocol by Jenkin et al. [9], fresh *Arabidopsis thaliana* leaves were cut from the mentioned genotypes and used as our samples. Tissue delipidation by chloroform, depolimerization by sodium methoxide, derivatization by N, O-Bis(trimethylsilyl)trifluoroacetamide (BSTFA), and GC-MS Analysis with an HP-5 capillary column. Software used: Enhanced MSD ChemStation E.02.00 SP2 by Agilent Technologies (Santa Clara, CA, USA). In conducting this research, certain modifications were made to the published protocols to accommodate the available equipment, conditions, and materials. While the overall methodology followed established procedures, necessary adjustments were implemented to ensure compatibility with the specific experimental setup. These adaptations were undertaken to optimize the experimental process and achieve reliable results within the given resources and constraints.

### 4.3. GC-MS Analysis

For the gas chromatography (GC) analysis, an HP-5 capillary column (Agilent Technologies, 7890A, USA) with dimensions of 30 m × 0.25 mm × 0.25 μm film thickness was utilized. The GC system was equipped with helium as the carrier gas, flowing at a rate of 1.5 mL/min. The oven temperature was programmed to increase from 150 to 300 °C at a rate of 3 °C/min. Split injection with a ratio of 1:10 was employed. The mass spectrometer (Agilent Technologies, 5975C, USA) was operated in scan mode, covering a range of 40–600 amu. Each sample was analyzed using the specified method.

### 4.4. SEM Imaging

The SEM was used to view epicuticular wax crystallization patterns. Inflorescence stem segments were collected from *Arabidopsis* wild-type (Col-0) and RabA2b overexpressing plants after four weeks of growth. The bottom part of the stems was cut into 0.5 cm long segments and then split in half vertically. The fixation method utilized fume fixation, where the stem segments were exposed to an 8% paraformaldehyde and 4% glutaraldehyde solution in water without direct contact for 24 h. Subsequently, the samples were fixed for 12 h in osmium tetroxide without direct contact. Afterward, the samples were dried under vacuum and coated with a 4 nm layer of iridium [41].

After capturing SEM images of various stem surface areas, the identification and quantification of long and thin crystal shapes were conducted using the ImageJ computer software (version 1.53, National Institutes of Health, Bethesda, MD, USA). For the purpose of this study, crystal shapes longer than 1.5 μm and thinner than 0.4 μm were considered as long and thin crystals. However, it should be noted that the measurement accuracy was limited due to the three-dimensional nature of the images. As an approximation approach, each image was divided into four regions, and the long and thin crystal shapes within each region were marked using the multi-point tool in ImageJ. This tool not only marked the crystal shapes but also counted the points created, providing a quantitative measure. Subsequently, the collected data were analyzed using a *T*-test for statistical analysis. Sequential zoom-in SEM micrographs of the stem epicuticular topography and the diversity of the wax crystals (including long-hair-like structures) can be visualized in Supplemental Figure S1.

## 5. Conclusions

The work presented here demonstrates that *RabA2b* contributes to protection against water loss via modifications of the cuticle’s biochemical composition and structural features. By promoting the accumulation of phenylpropanoid- and indole-derived metabolites, such as ferulate, coumarate, and indole-3-carboxaldehyde, and enhancing the formation of hair-like epi-cuticular wax crystals, *RabA2b* OE plants exhibit improved drought tolerance and potentially increased pathogen resistance. These findings not only provide insights into the molecular mechanisms by which vesicle trafficking regulates cuticle-mediated stress responses but also suggest a practical strategy to enhance crop resilience. In the context of the ongoing climate change crisis, which is predicted to increase the frequency and severity of drought and other water-related stresses [42]. Understanding and harnessing *RabA2b*-mediated cuticle modification could inform the development of tools to improve maintenance of water homeostasis in crops and to sustain productivity under adverse environmental conditions.

## Figures and Tables

**Table 1:** Average fold change in Cutin polyester monomers in *RabA2b* overexpressing (*OE*) transgenic plants compared with wild type (*WT*).

Compound Name	RT [min]	Average Fold Change 6.4 OE/WT ^1^	Average Fold Change 11.4 OE/WT ^1^
Coumaric acid	11.39	1.36 *	1.59 *
1H-indole-3-carboxaldehyde	11.78	1.27 **	1.28 *
16:0 FA	15.01	0.96	0.99
Ferulic acid	15.80	1.55 ****	1.55 ***
18:2 FA	19.72	1.02	1.01
18:3 FA	19.90	1.08	1.02
Sinapic acid	20.35	1.04	1.12
18:0 FA	20.65	1.08	1.04

^1^ Data represent mean values from three independent experiments, each including four to five biological replicates per line. Statistical significance was calculated using the one-way ANOVA test (Student’s *t*-test). Asterisk (*, **, ***, ****) represents significant values of *p* ≤ 0.05, *p* ≤ 0.01, *p* ≤ 0.001 or *p* ≤ 0.0001, respectively.

## Data Availability

No new data were created or analyzed in this study.

## Supplementary Materials

The following supporting information can be downloaded at: https://www.mdpi.com/article/10.3390/plants15030408/s1. Figure S1: SEM micrographs of the epicuticular topography in Arabidopsis stems. Representative SEM micrographs of Arabidopsis (wild type Col-0) inflorescence stem. The stem samples were vapor fixed, air-dried, coated with ~5 nm iridium, and visualized by SEM as described by Shemesh et al. (2017) [23]. Presented is a series of micrographs that were captured in a zoom-in sequential manner, indicated by red arrows, which point to the enlargement segment of the area in the red boxes. In the bottom two images, note the various sizes and shapes of the wax crystals, including hair-like, columnar-shaped rods, vertical plates, dendritic, and umbrella-like structures.
