# Supplementary material for: Chemical and Ultrastructural Changes in the Cuticle Observed in RabA2b Overexpressing Plants
Source: Plants (Basel). 2026 Jan 29;15(3):408. doi: 10.3390/plants15030408 (PMC12899485; doi:10.3390/plants15030408)
Supplement: Supplementary file 1 [file plants-15-00408-s001.zip › plants-4114886-supplementary.pdf]

Supplementary Material

Chemical and ultrastructural changes of the cuticle are observed in RabA2b overexpressing plants

Opal Bechar[1,2], Sanaa Musa[1,2], Boris Fichtman[3], Ifat Matityahu[1] and Yehoram Leshem[1,2*]

[1] MIGAL – Galilee Research Institute, Kiryat Shmona, Israel
[2] Faculty of Sciences and Technology, Tel-Hai College, Upper Galilee, Israel
[3] Azrieli Faculty of Medicine, Bar-Ilan University, Safed, Israel.
* Correspondence: yoril@migal.org.il; Tel.: +972-(0)58-6334648

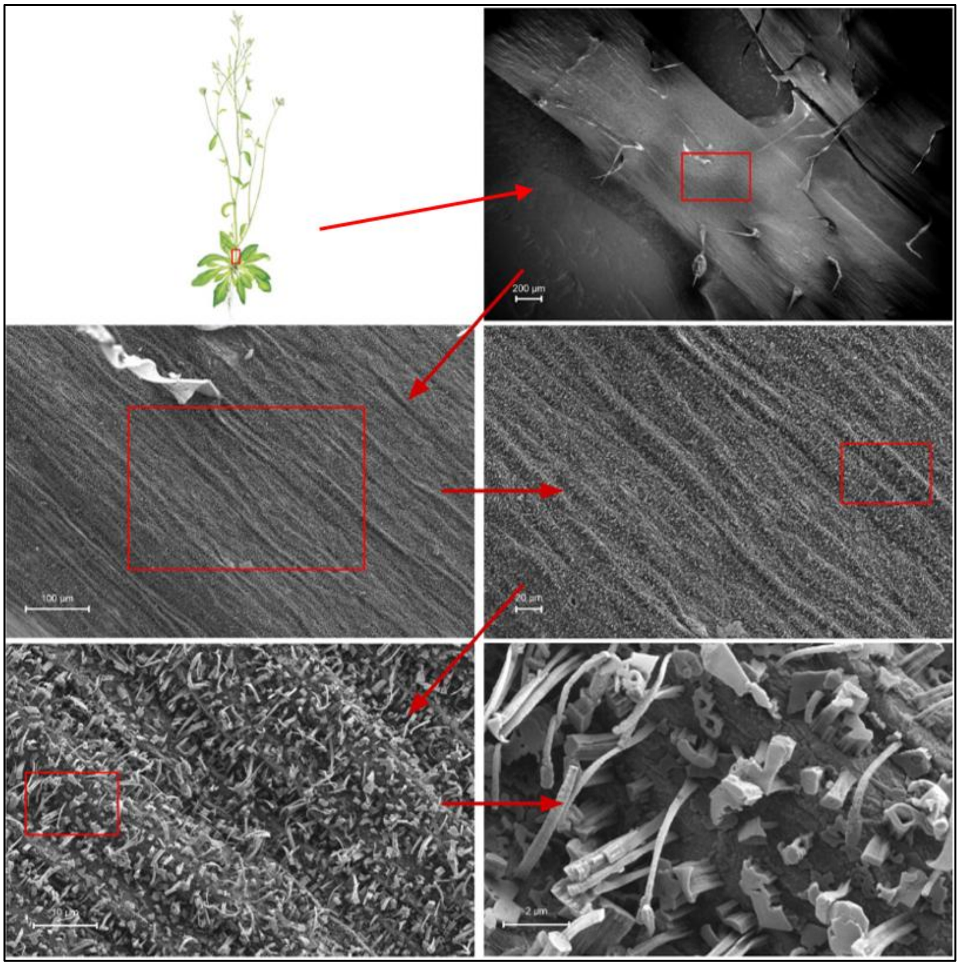

Figure S1 - SEM micrographs of the epicuticular topography in Arabidopsis stems. Representative SEM micrographs of Arabidopsis (wild type Col-0) inflorescence stem. The stem samples were vapor fixed, air-dried, coated with ~5 nm iridium, and visualized by SEM as described by Shemesh et al (2017)*(1)* . Presented is a series of micrographs that were captured in a zoom-in sequential manner, indicated by red arrows, which point to the enlargement segment of the area in the red boxes. In the bottom two images note the various sizes and shapes of the wax crystals, including hair-like, columnar-shaped rods, vertical plates, dendritic, and umbrella-like structures.

(1.) Shemesh, E., Hanf, B., Hagag, S., Attias, S., Shadkchan, Y., Fichtman, B., Harel, A., Krüger, T., Brakhage, A. A., Kniemeyer, O., and Osherov, N. (**2017**) Phenotypic and Proteomic Analysis of the Aspergillus fumigatus ΔPrtT, ΔXprG and ΔXprG/ΔPrtT Protease-Deficient Mutants, *Frontiers in Microbiology Volume 8 - 2017*.
